# The Wildlife–Livestock Interface as a Bidirectional Pathway for the Spread of ESBL-Producing *Escherichia coli*

**DOI:** 10.3390/ani16121859

**Published:** 2026-06-16

**Authors:** Margarita González-Martín, María Teresa Tejedor-Junco, Nerea C. Rosales-González, Juan Alberto Corbera

**Affiliations:** 1 Research Institute of Biomedical and Health Sciences, University of Las Palmas de Gran Canaria, Paseo Blas Cabrera Felipe “Físico” s/n, 35016 Las Palmas de Gran Canaria, Canary Islands, Spain; margaritarosa.gonzalez@ulpgc.es (M.G.-M.); juan.corbera@ulpgc.es (J.A.C.); 2 Clinical Sciences Department, University of Las Palmas de Gran Canaria, Paseo Blas Cabrera Felipe “Físico” s/n, 35016 Las Palmas de Gran Canaria, Canary Islands, Spain; 3 Servicio de Medicina Interna, Hospital Universitario de Gran Canaria Dr Negrín, C. Plaza Barranco de la Ballena, s/n, 35010 Las Palmas de Gran Canaria, Canary Islands, Spain; nrosgon@gobiernodecanarias.org; 4 Hospital Clínico Veterinario, Facultad de Veterinaria, University of Las Palmas de Gran Canaria, Campus Universitario de Arucas, s/n, 35413 Arucas, Canary Islands, Spain

**Keywords:** antimicrobial resistance, biosecurity, environmental reservoirs, ESBL-producing *Escherichia coli*, genomic surveillance, livestock, One Health, wildlife, wildlife–livestock interface

## Abstract

Antimicrobial resistance is a major global health challenge that requires a One Health approach integrating humans, animals, wildlife, food systems and the environment. Among resistant bacteria, extended-spectrum β-lactamase-producing *Escherichia coli* (ESBL-producing *E. coli*) is particularly relevant because it is widely distributed across hosts and ecosystems, may carry mobile resistance genes and is commonly used as an indicator for antimicrobial resistance surveillance. This narrative review examines the occurrence, characteristics and transmission dynamics of ESBL-producing *E. coli* at the wildlife–livestock interface, with emphasis on its public health relevance and strategies for mitigation and control. The reviewed evidence indicates that livestock, wildlife and environmental matrices can be interconnected reservoirs of resistant *E. coli* and resistance genes. Transmission should not be interpreted as a simple linear process from livestock to wildlife or humans but rather as a bidirectional and ecological phenomenon shaped by antimicrobial use, farm management, biosecurity, wildlife ecology, environmental contamination and mobile genetic elements. Wildlife may function as a sentinel, reservoir or disperser of resistant bacteria, although detection alone does not demonstrate direct transmission. Integrated surveillance combining livestock, wildlife, food-chain and environmental sampling, supported by genomic analysis, is essential to clarify transmission pathways and guide effective control measures.

## Simple Summary

Antibiotic resistance is a growing problem that affects people, domestic animals, wildlife and the environment. One important example is *Escherichia coli*, a common gut bacterium that can acquire mechanisms allowing it to survive treatment with important antibiotics. Livestock may carry these resistant bacteria, but wildlife can also become exposed through contaminated water, soil, manure, farms, waste and other human-influenced environments. This review summarizes the current knowledge on resistant *Escherichia coli* at the wildlife–livestock interface, with special attention to how these bacteria may move between farm animals, wild animals and the environment. The available evidence shows that transmission is not always one-way from livestock to wildlife or from animals to humans; rather, it is a complex and bidirectional process involving animals, people, food-production systems and environmental reservoirs. Wildlife can act as a sentinel of environmental contamination and, in some cases, may contribute to the spread or reintroduction of resistant bacteria into farms. Understanding these connections is important for society because it can help improve surveillance, farm biosecurity, responsible antibiotic use and coordinated control measures to protect animal, human and environmental health.

## 1. Introduction

Antimicrobial resistance (AMR) is a global problem that must be combated with a comprehensive “One Health” approach [1,2,3]. Infections caused by multidrug-resistant (MDR) bacteria have become a global health challenge.

Bacteria can transfer resistance genes horizontally, including between different species. The set of genes that confer resistance to one or more antibiotic families can be transferred between pathogenic and non-pathogenic bacteria in humans and animals (both domestic and wild), as well as with environmental bacteria [4,5,6]. This evolution toward multidrug resistance is the result of complex bacterial events involving mutations and gene transfers between species via mobile elements [7]. The increasing prevalence of MDR or extensively drug-resistant (XDR) bacteria reinforces the need to develop surveillance studies to detect and control these strains [8].

As indicated in the “WHO—Global Action Plan on Antimicrobial Resistance” [9], this problem represents a drain on the global economy, with economic losses due to reduced productivity caused by diseases (in both humans and animals) and increased treatment costs. Although not explicitly mentioned, it is clear that the increase in antibiotic resistance affects the achievement of sustainable development goals (SDGs). The increase in animal infections caused by antibiotic-resistant bacteria makes it difficult to build sustainable food-production systems and therefore hinders the SDGs on health, poverty, food security and economic growth [10].

Wildlife has been identified as a driver of the spread of genes conferring resistance to clinically important antimicrobials [11,12,13]. Although theoretically wild animals are not exposed to clinically relevant antibiotics, the detection of MDR strains in wildlife is increasing considerably, reinforcing the need for studies focused on this issue [14,15]. Furthermore, monitoring these bacteria in wild animals has become an important surveillance tool, as it could reflect antimicrobial resistance in strains isolated from humans [16].

In recent years, increasing attention has been paid to multidrug-resistant Enterobacterales, particularly ESBL-producing, plasmid-mediated AmpC β-lactamase-producing and carbapenemase-producing strains, across clinical, community, animal and environmental settings [17,18,19,20]. Within this group, ESBL-producing *E. coli* has been proposed as a relevant and practical indicator for AMR surveillance [21].

The aim of this narrative review is to analyze the information on the incidence, characteristics and transmission dynamics of ESBL-producing *E. coli* between livestock and wildlife. The public health relevance of this interface and the strategies for mitigation and control are also discussed. We highlight the need to address this issue from a One Health perspective.

***Literature Search Strategy and Selection Criteria***. This article was designed as a narrative review. A literature search was conducted using PubMed, Scopus and OpenAlex to identify publications related to ESBL-producing *Escherichia coli*, antimicrobial resistance and the wildlife–livestock interface. Searches were performed mainly in English and combined terms related to the bacterial target, host species, transmission routes, public health implications, mitigation strategies and surveillance. The search focused primarily on publications published between 2020 and 2026, although earlier studies were also included when they were considered relevant, widely cited or necessary to provide background information.

A summary of the methodology used can be found in Table 1.

Other search terms included: “food-producing animals”, “cattle”, “pigs”, “swine”, “poultry”, “sheep”, “goats”, “horses”, “wildlife–livestock interface”, “farm environment”, “environmental reservoirs”, “One Health”, “public health”, “foodborne transmission”, “direct contact”, “farm workers”, “biosecurity”, “antimicrobial use”, “antimicrobial stewardship”, “integrated surveillance”, “genomic surveillance”, “whole-genome sequencing”, “plasmids”, “mobile genetic elements” and “*mcr*”. These terms were combined using Boolean operators according to the topic of each section.

Titles and abstracts were screened to assess relevance to the objectives of this review. Publications were included when they provided information on the occurrence, molecular characterization, transmission dynamics, public health relevance, biosecurity implications, antimicrobial-use reduction or surveillance of ESBL-producing *E. coli* or related antimicrobial-resistant bacteria in wildlife, livestock, food products, humans or environmental matrices. Original research articles, reviews and relevant institutional documents were considered. Additional references were identified by screening the reference lists of selected publications. Publications were excluded when they were outside the scope of the wildlife–livestock–human–environment interface, did not address antimicrobial resistance or ESBL/AmpC-producing bacteria, lacked sufficient methodological information, or were not relevant to the objectives of this review. Because this was a narrative review, no formal meta-analysis or quantitative synthesis was performed.

## 2. ESBL-Producing *E. coli*

*Escherichia coli* is the bacterial species most frequently described in antibiotic resistance studies in wildlife [13]. Most animal species, as well as humans, carry *E. coli* in their intestinal tract. Most *E. coli* strains are commensals that inhabit the intestinal tract of humans and animals. However, *E. coli* also remains one of the most frequent causes of several common bacterial infections in humans and animals. In humans, it is an important opportunistic pathogen associated with severe sepsis and urinary tract infections, among other hospital-acquired infections [22].

In animals, *E. coli* can also cause various infections, with respiratory infections being particularly relevant in birds [22]. Vidal et al. [23] observed that *E. coli* was the most common microorganism recovered from necropsies of raptors with clinical signs of septicemia or respiratory disorders, finding MDR in 71% of *E. coli* isolates.

In livestock, infections caused by pathogenic strains of *E. coli* cause significant economic losses, affect animal welfare, and have repercussions for public health due to the transmission of certain serotypes to humans. In livestock, diseases associated with pathogenic *E. coli* include diarrhea in piglets, calves and lambs, urinary tract infections and mastitis [24,25,26,27].

β-Lactamases are bacterial enzymes capable of hydrolyzing the β-lactam ring of β-lactam antibiotics to render these compounds inactive [28]. To facilitate understanding of their structural, functional, and clinical relevance, β-lactamases are classified using two main schemes: the Ambler molecular classification [29,30] and the Bush–Jacoby–Medeiros (BJM) functional classification [31]. The latter system is particularly valuable in clinical microbiology, as it aids in predicting antimicrobial susceptibility and guiding therapeutic decisions.

The most epidemiologically significant enzymes in this context are extended-spectrum β-lactamases (ESBLs), which have become widely disseminated worldwide. These enzymes can hydrolyze penicillins, extended-spectrum cephalosporins and aztreonam. They are included in Group 2 in the BJM classification. Although ESBLs share common biochemical properties with regard to the hydrolysis of broad-spectrum beta-lactam antibiotics and inhibition by clavulanate, the genes encoding these enzymes are diverse in nature and can be grouped into several families. Table 2 summarizes the ESBL families described in *E. coli*.

The genetic flexibility and adaptability of *E. coli* to constantly changing environments allow it to acquire a large number of antimicrobial resistance mechanisms. CTX-M type beta-lactamases are the most frequently isolated ESBLs in Enterobacteriaceae species from human and animal samples [22,32,33].

**Table 2:** ESBL families described in *E. coli*.

ESBL Family	Nomenclature/Origin	Main Characteristics in ***Escherichia coli***	Reference
TEM	Derived from TEM-1 and TEM-2 β-lactamases (“Temoniera”). Common variants include TEM-3, TEM-10, TEM-52.	Class A ESBLs generated by point mutations. Hydrolyze penicillins and third-generation cephalosporins. Usually inhibited by clavulanic acid. Historically among the first ESBLs detected in *E. coli.*	[34]
SHV	Originated as chromosomally encoded β-lactamases of *K. pneumoniae*. “Sulfhydryl Variable”. Common variants: SHV-2, SHV-5, SHV-12.	Class A ESBLs with mutations expanding activity toward cefotaxime and ceftazidime. Frequently plasmid-mediated and widely disseminated among Enterobacterales.	[35]
CTX-M	“Cefotaximase-Munich”. Includes CTX-M-1, CTX-M-2, CTX-M-8, CTX-M-9, and CTX-M-25 groups.	Currently the predominant ESBL family worldwide in *E. coli*. Strong hydrolytic activity against cefotaxime. Associated with conjugative plasmids and community/hospital dissemination. Derived from chromosomal genes of *Kluyvera* spp.	[34,36]
OXA	“Oxacillinases”. Includes OXA-1, OXA-10, OXA-48 variants.	Class D β-lactamases. Some variants exhibit an ESBL phenotype. Hydrolyze oxacillin and some cephalosporins. Less susceptible to inhibition by clavulanic acid.	[34,37]
PER	“*Pseudomonas* Extended Resistance”. Example: PER-2.	Less common ESBLs in *E. coli*. Hydrolyze broad-spectrum cephalosporins. More frequently identified in *Pseudomonas aeruginosa* and *Acinetobacter* spp.	[34,38]
VEB	“Vietnam Extended-spectrum β-lactamase”. Example: VEB-1.	Plasmid-mediated ESBLs able to hydrolyze third-generation cephalosporins and aztreonam. Occasionally reported in *E. coli.*	[34,39]
GES	“Guiana Extended Spectrum”. Examples: GES-1, GES-5.	Some variants behave as ESBLs, while others evolved into carbapenemases. Frequently associated with mobile genetic elements and multidrug resistance.	[34,40]
BES	“Brazilian Extended Spectrum”.	Rare ESBL family described in Enterobacterales. Confers resistance to broad-spectrum cephalosporins.	[34]
TLA	“Tlahuicas”. Example: TLA-1.	Rare ESBL initially described in Latin America. Associated with transferable plasmids in Enterobacterales.	[34,39]
SFO	Derived from *Serratia fonticola*. Example: SFO-1.	Plasmid-mediated class A cefotaximase. Hydrolyzes broad-spectrum cephalosporins and often coexists with other resistance genes.	[34,41]
CMT	Complex mutant derived from TEM-1	TEM variants that are resistant to inhibition by clavulanate and sulbactam and also exhibit an ESBL phenotype.	[42]
BEL	Belgium extended β-lactamase. BEL-1 in *E. coli*	Preferentially hydrolyzes ceftazidime and aztreonam compared with cefotaxime.	[43]

There are several reasons for using ESBL-producing *E. coli* as a relevant and simple indicator for AMR surveillance. Among these, it is worth highlighting the possible connection between antibiotic use in farm animals and clinical cases in humans and the correlation between interventions focused on decreasing the exposure to antibiotics in humans and animals and the decrease in occurrence of ESBL-producing *E. coli* [21]. In addition to this, ESBL-producing bacteria are resistant to critically important antimicrobials including most beta-lactams, so treatment with last-line antimicrobials may be required.

## 3. ESBL-Producing *E. coli* in Wildlife

Antibiotic resistance can be a useful indicator of human influence on wildlife exposure to resistant bacteria. Animals in the wild may acquire these bacteria from treated water, farms, landfills, and other anthropogenically impacted environments [44,45,46,47,48,49]. ESBL-producing *E. coli* isolates have been reported in a wide range of wildlife species, particularly in wild birds [33,50,51,52,53,54,55,56,57,58,59]. In scavenging birds, carcasses of domestic animals available in the field represent an essential food resource, but they may also constitute an important source of exposure to veterinary drugs. The presence of antibiotic residues in carcasses ingested by necrophagous birds can contribute to the selection of antibiotic-resistant bacteria in their digestive tract [58,60,61,62].

ESBL-producing *E. coli* has also been described in several groups of wild animals. Among carnivores, isolates have been reported in large species, such as captive lions and tigers [63], and in mesocarnivores, including Iberian wolves [64,65], Iberian lynx [65,66], coyotes [67], raccoons [68,69] and foxes [11,70,71]. Wild ungulates and other ruminants, such as red deer, roe deer and buffalo, have also been identified as carriers of ESBL-producing *E. coli* [72,73,74,75,76,77,78,79]. These species may be raised on farms or share pastures with domestic ruminants, which can facilitate the transmission of microorganisms. ESBL-producing *E. coli* has also been detected in captive camels (*Camelus dromedarius*) in the Canary Islands [80]. In Europe, wild boar (*Sus scrofa*) is considered a model species for studying antimicrobial resistance at the wildlife–livestock interface [81], probably because of its high population and the potential implications of contact with domestic pigs [65,76,79,82,83,84,85,86,87].

Other wildlife groups [65,76,79,81,82,83,84,85,86,87] have also been investigated as potential reservoirs of ESBL-producing *E. coli*. These bacteria have been found in non-human primates, including baboons, chimpanzees, green monkeys and gorillas [88,89,90]. Rodents have also been studied as carriers of ESBL-producing *E. coli* and as potential reservoirs for livestock [91,92,93,94,95,96,97], with reported prevalences ranging from 1.5% to 5% and *Rattus norvegicus* being the most frequently cited species. Other small mammals, such as hedgehogs [53], coatis [98] and badgers [71], have also been described as reservoirs. The detection of ESBL-producing *E. coli* in bat fecal samples [65,99,100,101,102] is particularly relevant because of bats’ longevity, ecological adaptability and capacity for long-distance flight. In addition, bat guano is commonly used in agriculture as a biofertilizer, which may raise public health concerns.

Regarding semi-aquatic or aquatic species, ESBL-producing *E. coli* has been described in wild fish from the Atlantic Ocean [103] and the Mediterranean Sea [104], as well as in minks, otters and turtles [105,106,107]. In addition, a CTX-M-15-producing *E. coli* isolate was obtained from a captive dolphin in Portugal [108].

## 4. ESBL-Producing *E. coli* in Livestock

In this review, the term “livestock” is mainly used to refer to domesticated farm animals, particularly food-producing species. In most countries, this category usually includes cattle, sheep, goats, pigs, horses and donkeys, although in some regions species such as llamas, alpacas, camels, buffalo, reindeer or yaks may also be included. Poultry is considered separately in some epidemiological and production contexts because intensive poultry systems, hatcheries, flock management and processing chains may create specific conditions for the occurrence and dissemination of ESBL-producing *E. coli*. However, this distinction should be interpreted as operational rather than as evidence of epidemiological isolation. Poultry farms may be connected to other animal production systems through shared workers, equipment, feed, water sources, manure, waste, rodents, insects, wild birds or environmental runoff. Similarly, farmed amphibians, such as frogs, should not be regarded as isolated systems, since frog farms may have direct or indirect contact with poultry or other livestock production systems, especially when facilities are located close to each other or share water, drainage, personnel or environmental reservoirs. Therefore, the wildlife–livestock interface should be understood as a network of contacts among different food-producing animals, wildlife and the environment, rather than as strictly separated animal categories.

Given their public health implications, the presence of ESBL-producing bacteria in farm animals has been analyzed in several studies. In a recent review, the prevalence of ESBL-producing *E. coli* in healthy food-producing animals in different countries was compared. With some exceptions (UK and The Netherlands), they found that prevalence was higher in pigs than in cattle. Some countries, like Thailand (37%), China (44%), Portugal (49%), Germany (62%) and, especially, Lebanon (98%) presented very high prevalences in pigs [22].

Subsequent studies have also shown substantial variability in the prevalence of ESBL-producing *E. coli* among livestock species and geographical regions. In South Korea, Song et al. [109] found marked differences between pigs and cattle, with prevalences of 69.5% and 7%, respectively. A similar pattern was reported in livestock from Reunion Island, where Miltgen et al. [110] described a prevalence of 18.6% in swine compared with 1.6% in cattle. High prevalence has also been observed in pigs in Hungary, where Balázs et al. [111] reported a value of 72%. However, this pattern is not consistent across all regions. In Guadeloupe island, Gruel et al. [112] found a higher prevalence in beef cattle (14.7%) than in pigs (7.3%), whereas Chai et al. [113] did not detect ESBL-producing *E. coli* among swine isolates in Malaysia. In contrast, a study conducted in Italy reported similar prevalences in cattle and pigs, with values of 29.4% and 27%, respectively [114].

Studies in other livestock species are less frequent, but they indicate that ESBL-producing *E. coli* can also occur in small ruminants and equids. In sheep, pandemic blaCTX-M-8-producing ST224 *E. coli* has been described in Brazil [115], and Benavides et al. [91] reported a prevalence of 7.5% in sheep from small-scale farms in Chile. In the same study, goats (n = 6) and horses (n = 60) were also examined, but no ESBL-producing *E. coli* was detected. Marked differences between healthy cattle and sheep have been reported in Algeria, with prevalences of 17% and 1%, respectively [116]. In Portugal, 5.5% of sheep were identified as carriers of ESBL-producing *E. coli* [117], while in Tanzania a prevalence was found in both sheep and goats from different farms [118]. Goats carrying ESBL-producing *E. coli* isolates have also been described in Iran [119]. In diarrheic lambs, Shi et al. [120] recovered 28 *E. coli* isolates from 21 animals, of which 53.6% were ESBL producers. Equids may also act as carriers: approximately 21% of farm horses were positive in Israel [121], and ESBL-producing *E. coli* isolates were detected at a high frequency (53.5%) in samples from racehorses in Turkey [122].

## 5. The Wildlife–Livestock Interface

The wildlife–livestock interface has been defined as “the physical spaces where wildlife and livestock species overlap in range and may interact, creating opportunities for direct or indirect contact that could result in pathogen transmission in either direction” [123]. Although this definition refers specifically to pathogen transmission, it is also applicable to the exchange of multidrug-resistant bacteria and antimicrobial resistance determinants. As previously highlighted, there is a marked imbalance between the large amount of data available on the prevalence of ESBL-producing *E. coli* in wildlife and livestock and the limited evidence regarding the actual transmission of these bacteria at the wildlife–livestock interface [7,67].

Several factors should be considered when assessing the risk of exchange of multidrug-resistant bacteria at this interface. These include the livestock species involved, the farming system, the wildlife species present in the geographic area, the type and frequency of contact between hosts, possible transmission routes, and the degree of human influence on the environment [124,125]. The definition of “wildlife” is also relevant, since different interpretations may modify the risk factors considered. According to the proposal by Jori et al. [126], wildlife would not include species that depend on humans for food or reproduction, such as feral animals, animals kept in zoological gardens or rehabilitation centers, or individual animals dependent on supplementary feeding stations. Nevertheless, many studies on antimicrobial resistance in wildlife include precisely these situations, particularly when assessing the effects of anthropogenic pressure on resistant bacteria circulation [11,63,67,127].

Antimicrobial resistance in wildlife can be considered an indicator of human influence on ecosystems. Wild animals may acquire resistant bacteria from treated wastewater, farms, landfills, contaminated water bodies, agricultural environments or other anthropogenically impacted habitats [44,45,46,47,48,49]. Most publications on wild birds have focused on migratory, waterfowl or other highly mobile species [35,46,49,69]; however, urban-associated birds have also been investigated because of their public health relevance and their frequent exposure to anthropogenic sources of antimicrobial resistance [12,38,39,50].

Transmission of multidrug-resistant bacteria between wildlife and livestock may occur through several routes. Indirect transmission, through shared water sources, feed, pasture, soil, manure, fomites or contaminated environments, is generally considered the most common pathway [25,58,128]. Direct interactions between wildlife and livestock may also occur in some systems, although they are usually less frequent and more dependent on local ecological and management conditions [129,130]. In scavenging birds, carcasses of domestic animals available in the field constitute an important food resource, but they may also represent a source of exposure to veterinary drugs. The presence of antimicrobial residues in livestock carcasses consumed by necrophagous birds may contribute to the selection of resistant bacteria in their digestive tract, with possible implications for wildlife conservation and environmental dissemination of resistance [58,60,61,62].

Most available information on multidrug-resistant bacteria in wildlife is still based on prevalence studies. However, the detection of resistant bacteria in wild animals does not necessarily demonstrate their ability to transmit these microorganisms to livestock, humans or other wildlife species. Important knowledge gaps remain regarding whether these bacteria survive long enough in wild hosts or in the environment, and whether they are present at sufficient infectious doses to cause colonization or infection in other hosts. Future research should therefore assess the capacity of wildlife to disseminate resistant bacteria of relevance to livestock or public health, while also identifying where and how wild animals acquire these bacteria. This is essential both for limiting the spread of antimicrobial resistance and for reducing potential adverse effects on threatened wildlife populations [131].

Several studies have investigated the potential exchange of resistant *E. coli* and resistance determinants at the wildlife–livestock interface, although direct evidence of transmission remains scarce. Liu et al. [67] studied the genetic relatedness of ESBL-producing *E. coli* in wildlife and cattle sharing a habitat using shotgun metagenomic sequencing. Although ESBL-producing bacteria were detected in feral swine, no direct evidence of transfer to grazing cattle was found. However, bacteria carrying other resistance genes, including genes conferring resistance to tetracyclines and macrolides, were present in both animal groups, suggesting that feral swine could theoretically contribute to the exchange of resistant bacteria or resistance determinants with cattle.

In South Africa, Van den Honert et al. [132] compared antimicrobial resistance patterns of *E. coli* isolates from co-grazing and non-co-grazing livestock and wildlife species. Their results suggested that antimicrobial-resistant *E. coli* and resistance genes may be exchanged between livestock and wildlife sharing grazing areas, although clear evidence of ESBL transmission was not demonstrated. Similarly, Kozak et al. [133] showed that wild small mammals trapped on farms were five times more likely to carry tetracycline-resistant *E. coli* than animals trapped in natural areas, supporting the influence of livestock-associated environments on wildlife exposure to resistant bacteria.

At the buffalo–cattle interface in Southern Africa, Mercat et al. [134] found differences in antimicrobial resistance among *E. coli* isolates from buffalo populations with and without contact with cattle. No resistant *E. coli* strains were isolated from buffalo without cattle contact, whereas resistance was detected in buffalo sharing areas with cattle. A narrow-spectrum beta-lactamase-producing *E. coli* isolate carrying TEM-1 was found in both cattle and the in-contact buffalo population. In contrast, Navarro-González et al. [135] found no correlation between antimicrobial resistance in *E. coli* isolated from free-ranging cattle and cohabiting wild ungulates, including Iberian ibex and wild boar, in a natural environment.

Other studies have highlighted the role of anthropogenic pressure in shaping bacterial exchange among humans, livestock and wildlife. In Uganda, Rwego et al. [136] analyzed *E. coli* transmission among mountain gorillas, livestock and humans. Gorillas living in anthropogenically influenced areas harbored *E. coli* strains genetically similar to those from livestock and humans, whereas isolates from gorillas that did not share a habitat with humans or livestock were genetically distinct. Although ESBL production was not investigated, this study illustrates how human and livestock proximity may influence bacterial exchange in wildlife populations.

More recent genomic approaches have provided stronger evidence for local circulation of ESBL-producing *E. coli* among domestic and wild animals. Using whole-genome sequencing, Hayer et al. [137] studied ESBL-producing *E. coli* transmission dynamics in livestock, dogs and wild rodents in Chile. Nearly identical clones, carrying similar antimicrobial resistance genes, heavy metal resistance genes and virulence-associated genes, were detected in dogs, cattle and sheep from the same farm, as well as in a dog and a wild rodent from a nearby farm located approximately 15 km away. These findings support the possibility that resistant clones and plasmids may circulate among livestock, companion animals and wildlife in agricultural landscapes.

Overall, current evidence indicates that the wildlife–livestock interface can contribute to the circulation of multidrug-resistant bacteria and resistance determinants, but the magnitude and direction of transmission remain difficult to establish. Wildlife may act as a reservoir, spillover host, sentinel or disperser of antimicrobial resistance depending on the species, habitat, degree of human influence and type of contact with livestock. However, the mere detection of resistant bacteria in wildlife should not be interpreted as proof of effective transmission to livestock or humans. Future studies should move beyond prevalence data and combine simultaneous sampling of livestock, wildlife and environmental matrices with genomic analysis, ecological information and epidemiological modeling. Such integrated One Health approaches are needed to determine whether resistance dissemination at the wildlife–livestock interface occurs through clonal transmission, plasmid exchange, shared environmental contamination or broader anthropogenic pressure.

## 6. Public Health Implications

Although this review focuses on ESBL-producing *E. coli*, selected examples involving other antimicrobial-resistant bacteria are mentioned when they illustrate shared transmission routes, biosecurity challenges or One Health mechanisms relevant to the wildlife–livestock interface.

### 6.1. Risks of Transmission to Humans Through Direct Contact or Food

The public health relevance of antimicrobial-resistant bacteria at the wildlife–livestock–human interface lies in their capacity to circulate among food-producing animals, humans and the environment [138]. Food-producing animals may contribute to the emergence, maintenance and dissemination of resistant bacteria, which can subsequently reach humans through contaminated food products, direct animal contact or environmental exposure [138]. In the case of ESBL-producing *E. coli*, livestock and food products represent relevant sources of concern, as human exposure may occur through contact with colonized animals or through the consumption or handling of contaminated food [139]. Similarly, human colonization or infection with multidrug-resistant bacteria may occur through foods of animal origin, including meat, milk and eggs, as well as through direct contact with animals [140].

Occupationally exposed populations, such as farmers, farm workers and slaughterhouse personnel, are particularly relevant because resistant bacteria may circulate between animals, farm environments and humans. Although most evidence at this interface concerns ESBL-producing Enterobacterales, studies in pig production have also shown that livestock-associated resistant bacteria can be detected in animals, barn air, abattoir environments and farm workers. Schmithausen et al. reported livestock-associated methicillin-resistant *Staphylococcus aureus* (MRSA) in pigs, barn air and humans, while evidence for ESBL-producing Enterobacterales transmission from pigs to humans was less conclusive. These findings illustrate that farm and slaughterhouse environments may act as relevant interfaces for the dissemination of antimicrobial-resistant bacteria, including ESBL-producing *E. coli* [141].

Foodborne exposure is a major route by which resistant bacteria from animals may reach humans [140]. Although ESBL-producing *E. coli* is the focus of this review, other foodborne pathogens illustrate the public health relevance of this pathway. In *Salmonella enterica*, antimicrobial-resistant serovars associated with poultry meat products have repeatedly been reported as examples of transmission or dissemination along the food chain. For instance, multidrug-resistant ESBL-producing *Salmonella enterica* serovar Infantis carrying *bla*CTX-M-65 has been linked to contaminated chicken meat and human infections, supporting the need for control measures at both livestock and food-chain levels [142]. Other serovars, including Typhimurium, Newport and Blockley, have also been described in food-producing animals carrying clinically relevant resistance determinants, such as mobile colistin resistance genes, highlighting that this phenomenon is not restricted to a single *Salmonella* serovar [143]. These examples reinforce the importance of integrated surveillance of resistant Enterobacterales across livestock, food products and humans.

The public health significance of ESBL-producing *E. coli* is further increased when these isolates also harbor resistance determinants against critically important antimicrobials, such as fluoroquinolones or colistin. The detection of mobile colistin resistance genes, including *mcr*, in bacteria from food-producing animals is particularly concerning because these determinants may be transferred between commensal *E. coli* and other foodborne bacteria [139,143].

Wildlife may also be relevant to human exposure but mainly as an indicator of environmental contamination as a potential indirect link between natural, livestock-associated and human-associated environments. For example, Plaza-Rodríguez et al. [79] detected ESBL-/AmpC-producing *E. coli* in wild boars, roe deer, wild ducks and geese in Germany, although the overall prevalence was low and the findings did not demonstrate direct transmission to humans or livestock. Similarly, Brendecke et al. [144] identified ESBL-producing *E. coli* and *Klebsiella pneumoniae* in black-headed gulls, including high-risk *E. coli* lineages such as ST131, ST38 and ST58, with virulence-associated genes. Together, these studies indicate that wildlife surveillance can help identify the environmental circulation of clinically relevant resistance traits, but such findings should be interpreted as part of a broader One Health risk framework rather than as direct evidence of wildlife-to-human transmission.

At the same time, transmission should not be interpreted as universally direct or unidirectional. Genomic One Health studies may identify shared resistance determinants or mobile genetic elements without demonstrating direct animal-to-human strain transmission. Nguyen et al. [145] reported distinct blaCTX-M-harboring *E. coli* strains and plasmids in food animals and humans with community-acquired urinary tract infections, suggesting the existence of evolutionary barriers to direct strain or plasmid transfer across host species. However, the same study identified common insertion sequence elements flanking *bla*CTX-M variants across different sample sources, supporting the potential dissemination of resistance genes within or between habitats.

Overall, current evidence indicates that humans may be exposed to antimicrobial-resistant and zoonotic bacteria through direct animal contact, animal-associated environments and contaminated foods. However, the relative contribution of each pathway depends on the bacterial species involved, the production system, hygiene and biosecurity standards, food-chain controls and local environmental conditions [139,145,146].

### 6.2. Public Health Implications and One Health Relevance of Antimicrobial Resistance

Antimicrobial resistance at the wildlife–livestock–human interface represents a public health concern because resistant bacteria and mobile resistance determinants can circulate among humans, animals, food products and the environment [146]. This problem is no longer restricted to healthcare settings, as ESBL-producing Enterobacteriaceae have increasingly been detected in the community, including in healthy humans, food-producing animals, companion animals, wildlife and environmental reservoirs [144,147,148].

Food-producing animals and food products are relevant exposure points within this system. ESBL-producing and multidrug-resistant *E. coli* have been repeatedly reported in cattle, pigs, poultry and their production environments [149], and resistant bacteria may reach humans through direct contact, contaminated food or environmental pathways [150]. The detection of ESBL/AmpC-producing *E. coli* and *K. pneumoniae* in food products during production, including broiler meat, further supports the importance of the food chain as a route of human exposure [151]. However, direct animal-to-human transmission should not be assumed in all cases, since genomic One Health studies have shown that human and animal isolates may belong to distinct lineages sharing resistance determinants or mobile genetic elements [145].

Wildlife should be interpreted as part of this broader environmental ecology of antimicrobial resistance. Wildlife may act as sentinels, reservoirs or dispersers of resistant bacteria across natural and human-associated ecosystems [79,149], and the detection of ESBL-producing *E. coli* and high-risk *K. pneumoniae* lineages in wild birds further supports their inclusion in integrated surveillance frameworks [144]. Nevertheless, as discussed above, wildlife carriage should be considered evidence of environmental circulation rather than direct proof of transmission to humans or livestock.

A One Health approach is therefore essential to assess antimicrobial resistance across interconnected human, animal, food-production and environmental reservoirs. At the local scale, integrated surveillance enables comparison of resistant bacteria, resistance genes and mobile genetic elements from humans, animals, food products and environmental samples, helping to identify shared sources or possible transmission routes [146,152]. At a global scale, this approach is also necessary because food trade, international travel and animal movements may facilitate the dissemination of resistant bacteria, plasmids and resistance genes across regions [151].

Mobile genetic elements are particularly important in this context. Plasmids, integrons, transposons and insertion sequences can mobilize resistance genes, such as *bla*CTX-M, across bacterial populations, host species and ecological compartments [148]. Therefore, surveillance should not be limited to bacterial species but should also include resistance genes, plasmid lineages and other mobile genetic elements that may connect animal, human and environmental reservoirs [139,151].

Overall, the available evidence supports integrated surveillance across livestock, wildlife, companion animals, food products, humans and environmental matrices as a core public health strategy [148]. Mitigation efforts should combine prudent antimicrobial use, improved hygiene and biosecurity, safe food-handling practices, environmental control and genomic surveillance within a coordinated One Health framework [139,151].

### 6.3. Potential of Wildlife as a Source of Reintroduction of Resistance into Controlled Environments

Wildlife may contribute to the reintroduction of antimicrobial-resistant bacteria into controlled or partially controlled environments when livestock, companion animals, wild animals and environmental matrices share water sources, soil, pasture, feed, manure or peri-farm spaces [149]. This is particularly relevant for farm biosecurity, which aims [149] to prevent the introduction of microorganisms from external sources such as humans, vehicles, companion animals, wild animals, rodents, water and feed [153]. Even when antimicrobial use, cleaning and disinfection are controlled, resistant bacteria or resistance genes may persist in the surrounding environment and re-enter farms through wildlife, contaminated water, manure, soil, dust, feed or bedding materials [149].

Several studies support the plausibility of this process at different spatial scales. Fuentes-Castillo et al. [56] provided genomic evidence that migratory and resident gulls in Patagonia carried multidrug-resistant CTX-M-producing *E. coli* belonging to international lineages, suggesting that birds may contribute to the long-distance movement of ESBL-producing clones [56]. Similarly, Brendecke et al. [144] detected ESBL-producing *E. coli* and *K. pneumoniae* in black-headed gulls from German conservation islands, including strains phylogenetically related to humans, livestock, food and environmental sources [144]. At a more local scale, Hayer et al. [137] identified nearly identical ESBL-producing *E. coli* clones and plasmids that may circulate among domestic, peri-domestic and wild animals in agricultural landscapes.

These findings indicate that wildlife should not be viewed only as a passive indicator of environmental contamination but also as a potential ecological bridge between external reservoirs and managed animal production systems [154]. However, this role should not be overgeneralized, as the detection of resistant bacteria in wild animals does not, by itself, demonstrate efficient transmission to livestock or humans [79]. Therefore, studies assessing reintroduction should combine wildlife sampling, livestock monitoring, environmental sampling and genomic analysis to determine whether resistance spreads through bacterial clones, plasmids or shared resistance genes [137].

From a control perspective, these findings support the reinforcement of perimeter biosecurity, restriction of wildlife access to feed and water, improved manure and waste management, monitoring of rodents and wild birds, and the incorporation of wildlife into antimicrobial resistance surveillance programs [149].

## 7. Strategies for Mitigation and Control

Mitigation of ESBL-producing *E. coli* at the wildlife–livestock interface requires coordinated actions that reduce selection pressure, limit environmental contamination and prevent the movement of resistant bacteria or resistance genes between farms, wildlife and surrounding ecosystems. Therefore, control strategies should combine antimicrobial stewardship, farm biosecurity, environmental management and integrated surveillance within a One Health framework.

### 7.1. Reduction of Antimicrobial Use in Livestock

Reducing antimicrobial use in livestock is a central component of AMR mitigation, since antimicrobial use in food-producing animals contributes to the selection and dissemination of resistant bacteria and resistance genes. Antimicrobial use in livestock is not limited to therapeutic treatment, as antimicrobials may also be used for metaphylaxis, prophylaxis and growth promotion [138]. Inappropriate and excessive antimicrobial use, particularly when used to compensate for poor hygiene or suboptimal management, increases selection pressure [154]. Therefore, reduction strategies should focus on eliminating non-essential antimicrobial use while preserving appropriate therapeutic treatment when animal health and welfare require it [155].

Antimicrobial stewardship in livestock should not be understood only as a reduction in total antimicrobial consumption. It should also include appropriate antimicrobial selection, dosage, treatment duration, veterinary prescription control, avoidance of unnecessary group medication, and restriction of prophylactic or growth-promoting uses [149,154]. Reliable antimicrobial-use data are also needed, because current estimates of antimicrobial consumption in animals are affected by limited standardization across countries and production systems. Farm-level recording, prescription monitoring and integration of antimicrobial-use data with AMR surveillance are therefore essential components of reduction policies [149].

Reduction of antimicrobial use should be accompanied by measures that decrease disease pressure. Poor biosecurity, low vaccination coverage, inadequate nutrition and suboptimal husbandry can increase disease occurrence and reliance on preventive antibiotic use [140]. Preventive strategies such as vaccination, improved hygiene, public awareness, good husbandry practices, genomic surveillance and antibiotic stewardship have been recommended as part of AMR mitigation [148]. Alternative approaches, including prebiotics, probiotics, phage therapy, vaccines, antimicrobial peptides and antimicrobial polymers, may help reduce dependence on antibiotics, although their effectiveness should be considered within broader prevention programs rather than as universally validated replacements [149].

Evidence from alternative production systems also suggests that antimicrobial reduction alone is insufficient. Musa et al. found lower levels of cefotaxime-, ceftazidime- and ciprofloxacin-resistant *E. coli* in organic broiler systems than in conventional systems but a higher prevalence of tigecycline-, azithromycin- and gentamicin-resistant *E. coli* in organic samples [155]. This indicates that resistant bacteria may persist or be introduced through environmental or production-chain sources even when on-farm antimicrobial use is limited. At the environmental level, reducing antibiotic use in animals is also relevant because antimicrobial residues and resistant bacteria can enter soil and water through animal waste and food-production systems. Thus, reducing antimicrobial use in humans and animals, regulating antibiotic distribution and prioritizing less environmentally persistent antibiotics are important measures to reduce AMR in environmental reservoirs [146].

Overall, antimicrobial reduction should be framed as an integrated One Health intervention combining prudent use, restriction of non-essential indications, disease prevention, improved husbandry, vaccination, environmental management and continuous surveillance [148].

### 7.2. Biosecurity Measures on Farms

Farm biosecurity is essential to reduce the introduction, persistence and spread of resistant bacteria within and between animal populations. However, biosecurity should not be presented as a stand-alone solution, since its direct effect on antimicrobial resistance, particularly in non-pathogenic bacteria, is not always directly evaluated [152]. Instead, it should be integrated with herd health management, hygiene, vaccination, environmental control, antimicrobial stewardship and surveillance [152].

External biosecurity should aim to prevent the entry of resistant bacteria into farms through animals, people, vehicles, feed, water, wildlife, rodents, birds, insects and contaminated environmental matrices [156]. In broiler production, external measures are specifically intended to prevent microorganisms from entering farms through vectors such as humans, vehicles, companion animals, wild animals, rodents, water or feed [153]. Internal biosecurity should reduce dissemination between animal groups, pens, houses and production stages. Measures such as quarantine, all-in/all-out management, controlled animal movements, visitor and vehicle management, cleaning and disinfection, protective clothing, hand hygiene and isolation of affected animals can reduce infectious disease pressure and, consequently, the need for antimicrobial treatment [152].

Environmental hygiene is particularly relevant at the wildlife–livestock interface. ESBL-producing *E. coli* has been detected in manure-amended soil, barn dust, feed mixers, animal feed, bedding material and water troughs, supporting the inclusion of manure management, dust control, feed hygiene, water hygiene and bedding management in farm biosecurity plans [149]. Wild birds, rodents and insects should also be considered in biosecurity planning, especially in open or semi-open systems, where contact between livestock, wildlife and environmental reservoirs is difficult to avoid [149]. Water and waste management are also central, since resistant microorganisms and antimicrobial resistance genes may enter soil, air, water and sediments through manure, agricultural waste, wastewater and other environmental hotspots [157].

Cleaning and disinfection remain important, but they should be applied through well-designed protocols rather than excessive or indiscriminate disinfectant use, because antimicrobial resistance genes and disinfectant-resistance genes may be genetically linked [152]. Food-chain hygiene is also relevant, as hygienic handling during slaughter, processing and product handling can reduce contamination with ESBL-producing *E. coli* and other antimicrobial-resistant bacteria [158]. Although this review focuses on ESBL-producing *E. coli*, experiences with other livestock-associated resistant bacteria, such as MRSA, reinforce the need for coordinated hygiene, infection-control and animal-contact measures in farms and related environments [147,158,159].

Overall, farm biosecurity should combine controlled animal movements, visitor and vehicle management, quarantine, all-in/all-out systems, cleaning and disinfection, pest and wildlife control, feed and water hygiene, manure management, isolation of infected animals and integrated AMR monitoring [149,152].

### 7.3. Importance of Integrated Surveillance Across Livestock, Wildlife and the Environment

Integrated surveillance is necessary to identify reservoirs, detect emerging resistance trends, evaluate control measures and guide antimicrobial-use policies. From a One Health perspective, surveillance should not be restricted to clinical settings but should also include farms, veterinary settings, food products, wildlife and environmental matrices such as soil, water, manure and wastewater [160]. Environmental surveillance is particularly important because the environment can act both as a reservoir of resistance genes and as a niche in which antimicrobial resistance may emerge, persist and disseminate under anthropogenic pressure [146].

Surveillance programs should be designed to distinguish between bacterial transmission and the movement of resistance determinants through mobile genetic elements. For this reason, integrated AMR surveillance should combine microbiological isolation, antimicrobial susceptibility testing, molecular typing, genomic analysis, ecological information and epidemiological modeling [149]. Whole-genome sequencing can help determine whether resistance dissemination reflects clonal transmission, plasmid exchange or shared mobile genetic elements. For example, Nguyen et al. did not find direct evidence of food-animal-to-human transmission of *bla*CTX-M-harboring *E. coli* strains or plasmids in Vietnam, but they did identify common genetic elements across habitats, illustrating the importance of interpreting genomic evidence carefully [145].

Wildlife should be included because wild animals may act as sentinels of environmental contamination and, in some contexts, as reservoirs or dispersers of resistant bacteria. Plaza-Rodríguez et al. showed that, despite low overall prevalence in selected wildlife species, isolates from wild animals may reflect resistance traits to high-priority antimicrobials such as third-generation cephalosporins, fluoroquinolones and colistin [79]. However, the detection of resistant bacteria in wildlife should not automatically be interpreted as evidence of efficient transmission to livestock or humans. Surveillance should therefore aim to determine whether wildlife is acting as a sentinel, spillover host, reservoir or active disperser of AMR [79].

Highly mobile species and farm-associated wildlife deserve particular attention. Genomic studies in gulls have shown ESBL-producing *E. coli* carrying clinically relevant *bla*CTX-M genes related to strains from environmental, companion animal or livestock sources, supporting the value of wildlife genomic surveillance for identifying long-distance dissemination routes [56]. At the farm scale, Hayer et al. detected nearly identical ESBL-producing *E. coli* clones in livestock, dogs and a wild rodent, supporting the inclusion of livestock, companion animals, peri-domestic animals and wildlife in local surveillance programs [137]. Food-chain surveillance should also be integrated, since globally traded food products may carry ESBL/AmpC-producing *E. coli* and *K. pneumoniae* harboring multidrug-resistance genes and plasmids related to those found in livestock-associated or human clinical sources [151]. Human, animal and environmental data should be interpreted jointly, as shown by studies detecting ESBL-producing Enterobacteriaceae in farmers, children, livestock and environmental samples in rural communities [148].

Properly designed monitoring programs require standardized sentinel organisms, sampling strategies, culture methods and antimicrobial susceptibility testing and genomic interpretation criteria. Lack of harmonization may lead to misinterpretation, underreporting or unnecessary responses [152,160]. Overall, integrated surveillance across livestock, wildlife, humans, food products and the environment should be considered a core mitigation tool because it supports identification of reservoirs, clarification of transmission pathways, implementation of targeted biosecurity measures and evaluation of antimicrobial-use reduction policies [152,160].

## 8. Conclusions and Future Perspectives

Antimicrobial resistance at the wildlife–livestock–human interface should be understood as a dynamic and bidirectional process rather than as a linear pathway from livestock to humans [149,157]. ESBL-producing *E. coli*, resistance genes and mobile genetic elements may circulate among food-producing animals, wildlife, humans, food products and environmental matrices, and interventions implemented in one sector may influence the others [146,149,152]. However, the detection of resistant bacteria or shared resistance determinants in different reservoirs should not be automatically interpreted as evidence of direct transmission. Genomic studies may identify shared mobile genetic elements without demonstrating direct animal-to-human or wildlife-to-livestock strain transfer [145].

Future research should therefore move beyond prevalence-based approaches and integrate epidemiological, ecological and genomic data to distinguish between clonal transmission, plasmid dissemination and broader environmental circulation of resistance genes [56,145,149]. Evidence of both clonal and plasmid-mediated circulation of ESBL-producing *E. coli* among livestock, dogs and wildlife in agricultural settings reinforces the need to consider domestic animals, peri-domestic species and wildlife as interconnected components of the same epidemiological system [137]. Whole-genome sequencing and, when feasible, long-read or hybrid sequencing approaches should be prioritized because they can clarify whether resistance dissemination is driven by bacterial clones, epidemic plasmids or shared genetic platforms [56,145,151,153].

The One Health approach provides the most appropriate framework for addressing this challenge, as it explicitly connects human health, animal health, wildlife, food systems and environmental quality [155]. Interdisciplinary research involving veterinarians, microbiologists, ecologists, epidemiologists, environmental scientists, food-safety specialists and public-health authorities is needed to identify the drivers of antimicrobial resistance, including antimicrobial use, infection-control practices, farming systems, waste management, sanitation, environmental contamination, wildlife ecology and food-chain dynamics [146,149,155,157].

Future monitoring programs should combine livestock sampling, wildlife surveillance, food-chain monitoring and environmental sampling of water, soil, manure, wastewater and farm-associated matrices [149,151,155]. Surveillance should include antimicrobial susceptibility profiles, resistance genes, plasmids, insertion sequences, transposons and other mobile genetic elements that may connect different reservoirs [145,151,155]. Wildlife should be incorporated into AMR monitoring programs as a potential reservoir, sentinel or disperser of resistant bacteria, particularly in species with high mobility, close contact with livestock or frequent exposure to anthropogenic environments [79,149,153]. Selective isolation may be especially useful in wildlife surveillance when the expected prevalence of resistant bacteria is low but resistance to critically important antimicrobials, such as third-generation cephalosporins, fluoroquinolones or colistin, is of particular concern [79].

Control programs should combine prudent antimicrobial use, reduction of unnecessary prophylactic and growth-promoting uses, improved husbandry, vaccination, farm biosecurity, hygiene, pest and wildlife control, safe manure management and protection of water sources [149,155,160]. Their effectiveness should be evaluated through continuous surveillance, because antimicrobial resistance may persist or re-enter controlled systems through environmental reservoirs, mobile genetic elements, wildlife, food products or animal movements [56,149,151,155]. Standardized sampling strategies, harmonized laboratory methods and comparable antimicrobial susceptibility interpretation criteria are essential to generate reliable data and avoid underreporting, misinterpretation or disproportionate responses [79,155,160].

In conclusion, recognizing the bidirectional and ecological nature of antimicrobial resistance is essential for designing effective mitigation strategies at the livestock–wildlife–environment interface [149,155]. Future policies should prioritize integrated One Health surveillance, genomic epidemiology, environmental risk assessment and targeted farm-level interventions to reduce the emergence, persistence and reintroduction of resistant bacteria in animal production systems [56,149,155,160].

Local and simultaneous sampling of livestock, companion animals, wildlife and the farm environment should also be encouraged, as broad spatial comparisons or studies restricted to a single population may miss transmission events occurring at the farm level [56].

Monitoring programs should also collect data on antimicrobial use, animal movements, farm hygiene, biosecurity practices, manure management, water sources, wildlife access and food-production practices, because these variables are necessary to identify critical control points [149,155,160].

## Figures and Tables

**Table 1:** Some of the search strategies used in this narrative review.

Database	Search Strategies	Results
OPENALEX	ESBL and *Escherichia coli* and livestock and wild animals	21
	ESBL and *Escherichia coli* and farm animal and wild animals	13
	ESBL and “*E. coli*” and farm animal and wild animals	28
Scopus	ESBL AND “*Escherichia coli*” AND livestock AND “wild animals”	22
	ESBL AND “*Escherichia coli*” AND “farm animal” AND “wild animals”	4
	ESBL “*Escherichia coli*” AND livestock AND (wild animals) OR (farm animals)	165
	(ESBL AND “*Escherichia coli*” OR *E. coli*) AND (livestock OR farm animals) AND (wild animals)	49
	ESBL AND “*Escherichia coli*” AND “farm animal”	72
PubMed	ESBL and “*Escherichia coli*” and “Wild Animals” AND “Livestock”	10
	“ESBL” and “*Escherichia coli* or *E. coli*” and “livestock or Farm animal” and “wild animals”	14
	ESBL and “*Escherichia coli*” and “Wild Animals”	50
	ESBL and “*Escherichia coli*” and “Livestock”	309
	ESBL and “*Escherichia coli*” and Wildlife	235

## Data Availability

No new data were generated or analyzed in this study. Data sharing is not applicable to this article.
